# Outrunning protein diffusion to the air–water interface in cryoEM

**DOI:** 10.1073/pnas.2516900122

**Published:** 2025-10-22

**Authors:** Anastasiia Gusach, Kasim Sader, Christopher J. Russo

**Affiliations:** ^a^Medical Research Council Laboratory of Molecular Biology, Cambridge CB2 0QH, United Kingdom; ^b^Protein Sciences, Structure & Biophysics, Discovery Sciences, AstraZeneca, Cambridge CB2 0AA, United Kingdom

**Keywords:** cryoEM, air–water interface, specimen preparation, structural biology, electron microscopy

## Abstract

Here, we report a series of measurements indicating that it is physically possible to thin and vitrify a specimen for electron cryomicroscopy (cryoEM) faster than proteins diffuse to the air–water interface. We achieved this by spraying picoliter volume droplets at speeds of hundreds of meters per second into a thin layer of liquid ethane coating the surface of a precooled specimen support. The droplets simultaneously collapsed and froze in microseconds into the amorphous phase as they landed on the surface. The atomic structure of the proteins was preserved and tomographic reconstructions of the vitrified specimens indicated adhesion to the interfaces was eliminated. Improved control of the final thickness of the specimen and the orientation distribution of the particles are now the limiting factors. This demonstration provides a basis for the development of specimen preparation methods and instruments that eliminate the detrimental effects of the air–water interface in cryoEM.

Successful structure determination by electron cryomicroscopy is limited by the ability to prepare and vitrify a suitable specimen for imaging with electrons. During specimen preparation for cryoEM, particles diffuse to and concentrate at the air–water interface as the specimen is thinned using blotting, followed by plunge-freezing within a few seconds (1, 2). This air–water interface exposure often leads to damage to the protein particles and destruction of the specimen (1, 3, 4). The problem is so severe that structures are often determined with tens of thousands of micrographs where more than 99% of the data is discarded to obtain the fortunate few particles that have survived. For many targets, extensive optimization, including thousands of micrographs, are needed to achieve even this. Two methods are commonly used to ameliorate the detrimental effects of air–water interface adsorption: addition of surface-active agents such as detergents/surfactants (5, 6) or using support films such as amorphous carbon (7), graphene oxide (8), or graphene (3, 9, 10). In both cases, the air–water interface becomes less accessible to the protein particles just prior to freezing, but no method has yet proved universal for any purified specimen; all require significant amounts of trial and error. For purposes of time-resolved cryoEM (11) and automated grid preparation (12), specimen vitrification apparatuses have been constructed that reduce the time between thinning and freezing the specimen from seconds, which is typical for blotting, to milliseconds using sprayed droplets of specimen. The fastest of these are droplets sprayed into liquid ethane directly (13), or onto grids submerged in liquid ethane (14). Reducing the time between blotting or droplet impact on a grid reduces the number of collisions with the air–water interface but no method has yet been shown to eliminate them completely.

Regardless of the preparation technique used, the final thickness of a cryoEM specimen should not exceed a few hundred Ångströms. Given this, contact with the air–water interface seems certain, unless one can somehow coordinate the thinning of the liquid water layer with the rapid reduction in temperature required to quench it into a glass. During standard plunge freezing, the aqueous specimen comes into contact with liquid ethane at 90 K and a liquid–amorphous solid transition front propagates through the water. But how fast does this occur? If we take the thermal diffusivity of water as a guide (10^−4^ cm^2^/s) (15), we can compare it to the diffusion constant of a 500 kDa protein (10^−6^ cm^2^/s); the water solidifies two orders of magnitude faster than the protein diffuses. Furthermore, a droplet of water can be thinned at rates of 100 m/s which far exceeds the diffusion of proteins. Droplets can have a starting diameter from millimeters to a fraction of a micrometer (16, 17). Therefore, it is physically possible for freezing to outrun the molecules on their journey to destruction at the air–water interface, if only we could thin and freeze the water just fast enough and in a precisely coordinated sequence in time.

So how to thin and almost simultaneously vitrify a specimen of protein? Here, we sprayed small droplets at high speed onto cryogenically cooled EM grids coated in a thin layer of liquid ethane (Fig. 1*A*) and were able to completely interrupt diffusion of the particles to the air–water interface while still making a specimen suitable for structure determination by cryoEM. It is thus evident that methods for cryoEM specimen preparation which entirely eliminate interaction with the air–water interface are within the parameter space of water thinning and vitrification.

**Fig. 1.**
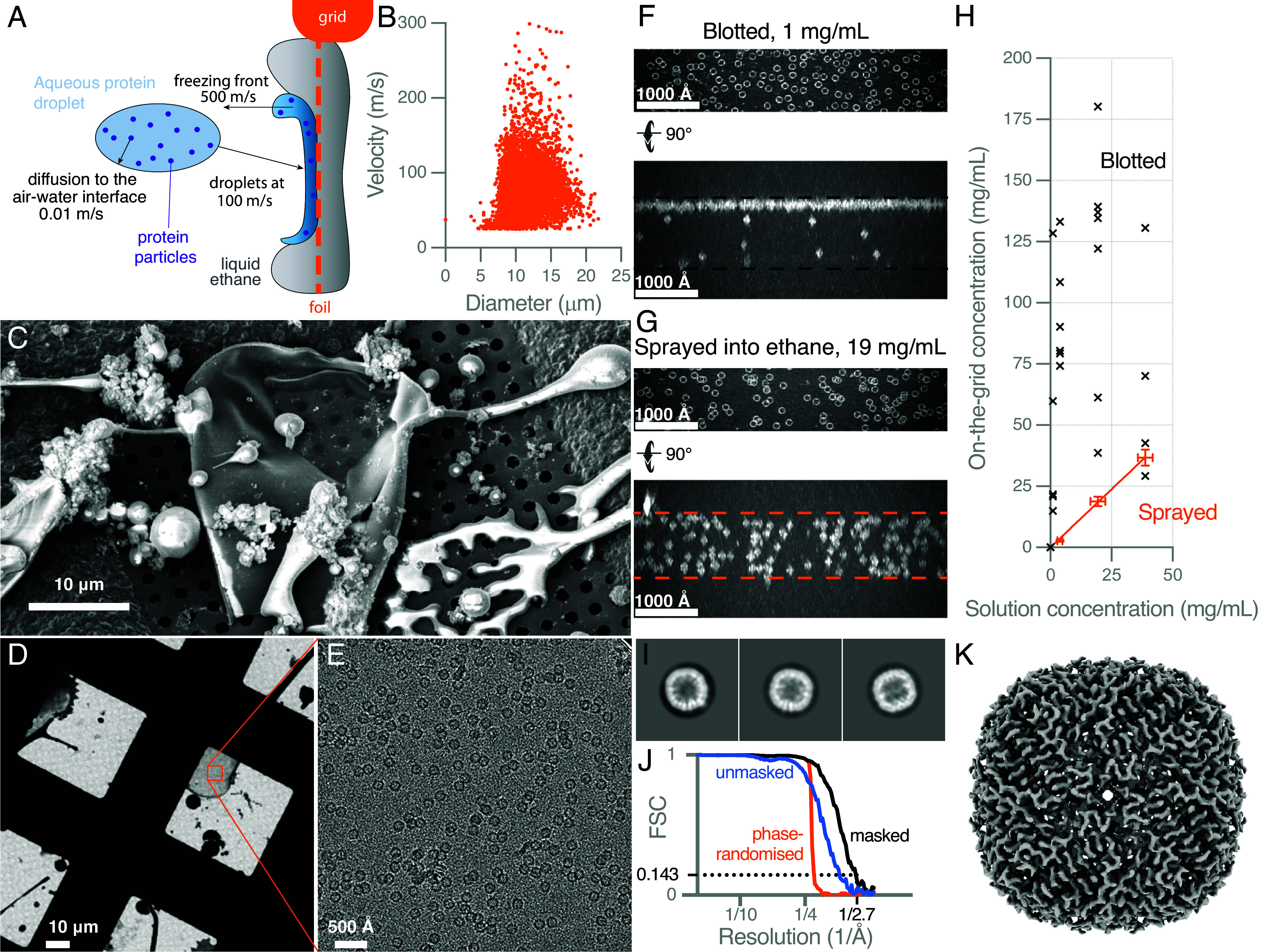
Thinning a cryoEM specimen faster than proteins diffuse using high speed droplets sprayed into a thin layer of ethane: an instrument was constructed to spray micrometer scale droplets of aqueous protein specimens at hundreds of meters per second onto a precooled EM grid coated in a thin layer of liquid ethane (*A*, see also Movie S1). The size and velocity distributions (*B*) of the droplets were measured using a high-speed camera. The droplets thin and spread on the grid as they are frozen and then the grids were imaged by scanning electron cryomicroscopy (*C*) and transmission electron cryomicroscopy (*D*). A magnified view of the spread out droplet in (*D*) is shown in (*E*), where individual apoferritin particles are distributed throughout the vitrified ice layer. 3D electron tomograms of specimens made with traditional blotting and plunge freezing (*F*) and high-speed droplet deposition (*G*) were reconstructed to determine the distribution of the particles in each case. This was repeated for several concentrations and then plotted against the concentration of the specimen measured in solution (*H*). A few hundred micrographs like the one in (*E*) were used to determine a 3D structure of apoferritin with a resolution of 2.7 Å (*I*–*K*).

## Results

A droplet sprayer vitrification device with programmable control of the grid position was constructed (Movie S1) that provided a defined pulse of droplets whose velocities were in the hundreds of meters per second, and whose diameters ranged from around 1 to 20 μm (Fig. 1*B*). The droplets were sprayed onto an electron microscope grid cooled to 91 K and coated in a thin layer of liquid ethane (order 10s of micrometers thick), as described in *SI Appendix*. Upon impact with the ethane coated surface, many of the droplets spread out and thin on the foil as they are vitrified (Fig. 1*C*). Because of the speed of the droplets hitting the thin layer of ethane, the spreading occurs within a few microseconds, (observed with a high-speed camera) and vitrification follows immediately. In the thin regions of large droplets, the water is amorphous, attached to the suspended foil and suitable for imaging using standard single particle cryoEM methods (Fig. 1 *D* and *E*).

To assess whether interaction with the air–water interface was interrupted, we collected low-dose tilt series to create 3D reconstructed maps of the specimens (tomograms). We then used these to compare the apoferritin concentrations in the test tube with those in the thin layer of amorphous water after freezing, for both standard plunge-freezing using blotting (Fig. 1*F*) and sprayed into a thin layer of ethane (Fig. 1*G*). Blotted specimens showed marked particle concentration at the air–water interface, resulting in highly variable concentrations on the grid (Fig. 1*H*). In contrast, sprayed specimens maintained a consistent concentration, matching the concentration in solution to within the error of the measurement (Fig. 1*H*). Each concentration was measured multiple times in solution and on multiple grids (≥3 per concentration); the SD for each is shown as the error in Fig. 1*H*.

Having verified that diffusion to the surface was interrupted, we collected a standard single particle dataset on the apoferritin specimen, selecting thin regions of large droplets similar to those shown in Fig. 1*C*–*E*. Reference-free 2D class averages show the secondary structure of the protein molecules (Fig. 1*I*), and the 3D reconstruction from 114,010 particles (Fig. 1*K*) had a resolution of 2.7 Å (Fig. 1*J*). The signal per particle (and thus the resolution) was limited by the thickness of the ice layer, which ranged from about 500 to 1,000 Å. This is thicker than what has been used for the highest-resolution structures of apoferritin, where the ice layer is just thicker than the protein complex adhered to an air–water interface (150 to 200 Å). Size exclusion chromatography experiments were performed before and after spraying several protein specimens onto the surface of a test tube (eppendorf) and these did not indicate any significant degradation caused by the spraying process (peaks remained sharp).

## Discussion

Complete control over the quality of single-particle cryoEM specimens taken from purified protein samples requires addressing three key factors: 1) eliminating air–water interface-induced or other forms of protein degradation during sample preparation, 2) controlling sample thickness, and 3) preventing orientation bias, all while being insensitive of the buffer composition. Currently, no method provides a general solution that addresses all of these requirements. Understanding the nature of each of the existing limitations is essential to eventually overcoming them. Here, we have established that specimens can be thinned and frozen in a way that prevents interaction with the air–water interface, thus solving the first of these problems. Since the particle concentration now matches the concentration in solution, the importance of starting with a homogenous concentrated specimen increases. Fortunately, even for a small protein (100 kDa) at modest concentrations (0.1 mg/mL), hundreds of particles can be expected per square micrometer in images (18). This fact and the speed of modern automated electron microscopes means that large datasets with hundreds of thousands of particle images remain feasible; improved methods for concentrating proteins in vitro without surface interaction will likely be of increasing interest for structure determination going forward. In the experiments here, the rate of thinning and the final thickness of the specimen are dependent on the size, velocity and fluid properties of the liquid droplets. Future work will create a system based on this advance that can control the final thickness of the specimen while still eliminating the surface interaction and maintaining a random orientation distribution. This would then turn structure determination by cryoEM from a trial-and-error art to a reproducible (and subsequently automated) process for any purified specimen.

## Materials and Methods

The detailed methods are described in *SI Appendix*. Briefly, purified specimens of apoferritin were sprayed onto precooled supports with a thin layer of liquid ethane on the surface. They were stored in liquid nitrogen until they were imaged using cryoEM. Data were collected and processed according to standard single-particle and tomography cryoEM methods.

## Supplementary Material

Appendix 01 (PDF)

Movie S1. High-speed camera recording of the full cycle of spraying and plunging for one clipped grid with a frame rate of 1.25 ms/frame

## Data Availability

Raw micrographs are deposited in the Electron Microscopy Public Image Archive (EMPIAR-13015) (19). All other study data are included in the article and/or supporting information.
