# Supplementary material for: Outrunning protein diffusion to the air–water interface in cryoEM
Source: Proc Natl Acad Sci U S A. 2025 Oct 22;122(43):e2516900122. doi: 10.1073/pnas.2516900122 (PMC12582301; doi:10.1073/pnas.2516900122)
Supplement: Supplementary file 1 — Appendix 01 (PDF) [file pnas.2516900122.sapp.pdf]

PNAS

Supporting Information for

Outrunning protein diffusion to the air-water interface in cryoEM

A. Gusach, K. Sader, C.J. Russo

Christopher J. Russo
E-mail: crusso@mrc-lmb.cam.ac.uk

This PDF file includes:

Supporting text
Legend for Movie S1
SI References

Other supporting materials for this manuscript include the following:

Movie S1

Supporting Information Text

Materials and Methods

High velocity droplet vitrification system. Both the plunging-spraying and imaging parts of the setup were assembled on an optical table (RS2000 Series Optical Table, 900x1800x305 mm, Newport) with isolator legs (S-2000 Series, Newport). A metal frame, isolated from the optical table, was constructed to accommodate electronics. The plunger was based on a linear motor with a stator (LinMot PS01-23x80F-HP-R) and a slider (LinMot PL01-12x420/380-HP), both mounted using a flange (PF02-23x50) on a custom-made holder that allowed sliding along a vertical post. The motor was controlled with a servo drive (LinMot A1100-GP-LC-0S-000) connected via a trailing cable (KS05-A-Fe/R-2). Another cable (DC01-X44-4m) was used for the connection with a laptop computer. A power supply (S01-72/500) was used to power the linear motor. An optical sensor was fixed to the same base rod above the motor, with its position adjusted to obtain the desired time delay from the start of the stroke. Ceramic replaceable-tip tweezers (Ideal-tek 2AMZ.SA.0) with a plastic stopper were attached to the slider using a custom-made dovetail brass mount. A custom-made, heated, nitrogen-ethane cup with an off-centre ethane compartment was placed on a custom-made, cylindrical holder which was secured to the optical table. An airbrush head unit (Iwata revolution M1) was placed on a custom-made mount allowing fine control of the tilt, horizontal-plane translation and vertical height adjustment. A continuous 360° rotation stage (CR1/M, Thorlabs), with an adapter plate (CR1A/M, Thorlabs) was used for the tilt angle adjustment of the airbrush. Two micrometer linear stages (XR50P/M, Thorlabs) allowed translation in the horizontal plane. A vertical translation stage (VAP10/M, Thorlabs) allowed vertical height adjustment. The airbrush was activated by pressurised argon gas, regulated by a pressure regulator with a range of 1 to 30 psi. The gas supply was delivered in short, reproducible pulses, with the timing of each pulse controlled by a solenoid valve. The valve was triggered by the movement of the plunger's sliding rod and controlled by a custom-made controller, which allowed for a minimal pulse durations of 1 ms.

Imaging setup. A high-speed camera (Phantom VRI VEO 1310S 36G M) with a manual focus 12X Zoom Lens (MVL12X12Z, Thorlabs) and a 2X extension tube (MVL20FA) was mounted on a vertical translation stage (VAP10/M, Thorlabs) placed on two crossed translation stages (TBBA1515/M, Thorlabs) for translations in the horizontal plane. A V-clamp mount (C1512, Thorlabs) fixed on a 1.5 inch post (P30/M, Thorlabs) on a magnetic base (PB1, Thorlabs) was used to support the lens. A ∼300 W white light source (Leica model EL6000) was coupled to liquid light guide and sent through a 400 nm long-pass filter (FGL400M, Thorlabs) fixed with a clamp (RA90/M, Thorlabs) to provide bright illumination for high-speed camera videos with exposure times in the microsecond range.

Droplet imaging and characterisation. The videos of the airbrush spray in the imaging plane were recorded with zoom magnification range of 4–14×. To improve the contrast for measuring the size and speed of the droplets, a trans-illumination configuration of the light source was used at maximum brightness such that the droplets appear dark on a bright backround. A snapshot of a calibration stage micrometer with 50 µm divisions (R1L3S1P, Thorlabs) was recorded after each video for precise scale determination. The frames of interest were selected in the PCC 3.8 software (Phantom) and exported as multipage .tif files. The Fiji-2 package (1) was used to determine the length of the tracks using the Ridge Detection plugin (2); the thickness of the track was taken as the diameter of the droplet. The velocities of the droplets were then determined using the known exposure time and the track length.

Preparing apoferritin specimens using high velocity droplets. Quantifoil 1.2/1.3 Mesh 400 grids were clipped in clip-rings with C-clips and were then plasma treated (30 s, argon:oxygen, 9:1, 38 W, using a Fischione model 1070) on top of a clean glass slide in batches of four, immediately before the spraying experiment. Grids were fixed in tweezers (Ideal-tek 2AMZ.SA.0), mounted into the plunger in its bottom position such that grids were placed into the liquid ethane. The angle between the spray axis and the grid in the plane parallel to the bench was approximately 45°. At least 30 seconds was allowed before the beginning of the spraying cycle to ensure thermal equilibrium between the clipped grid and the liquid ethane. A volume of 25 to 50 µL of protein was loaded into the Iwata M1 airbrush and the sprayer was activated by 30-60 ms pulses of argon (pressure 10-12 psi) synchronised with the start of the plunger stroke. The airbrush flow rate was adjusted using the back regulator knob which adjusts the position of the airbrush needle; it was set to mark 3 to 4 for the experiments. The plunger was programmed to move up for 100 ms with a sinusoidal trajectory of 200 mm, followed by a symmetric trajectory back down into the ethane (see Movie S1). The spray reached the grid at the point of the grid's maximum velocity (100 mm from the bottom position) on its way up. The airbrush nozzle was placed 2 to 3 cm away from the vertical path of the grid. High-speed camera videos of the spray hitting a grid were recorded for every experiment. The temperature of ethane in the cup was controlled at 90 ± 5K using a cryostat of the design in reference (3) where the ethane cup was offset to the edge of the plunging pool. Clipped grids were transferred into liquid nitrogen after the spray-plunge cycle, placed into grid storage boxes and then stored in liquid nitrogen before loading into an electron cryomicroscope for imaging. For tomography, apoferritin from equine spleen (Sigma) was gel filtrated by size-exclusion chromatography using an Agilent Bio SEC-5 HPLC Bio Column (Agilent) with Agilent Bio SEC-5 guard column (Agilent) equilibrated in the phosphate buffered saline (PBS) buffer pH7.4 and was used for all experiments. For structure determination, apoferritin was purified as previously described (4) and used at a nominal concentration of 50 mg/mL for the single-particle spraying experiment. Protein specimen concentrations in solution were measured by absorption spectroscopy at 280 nm. We estimated the specimen had approximately 10% ferritin contamination, so the extinction coefficient was taken as an average of the value for 9 parts apoferritin (0.72) and 1 part ferritin (3.6). Each specimen concentration was

measured at a series of at least five dilutions for each concentration, and the error was taken as the standard deviation between the measurements given the known amount of dilution. Two other methods were used to measure the concentration of the proteins in solution: direct detection FTIR spectroscopy and amido black staining (5). Both gave similar, but less reproducible results so the measurements using absorption at 280 nm are the ones reported in the figure.

Preparing apoferritin specimens using standard plunge freezing. For blotted grids, Quantifoil 1.2/1.3 Mesh 400 grids were plasma treated (30 s, argon:oxygen, 9:1, 38 W, using a Fischione model 1070). Apoferritin at the the several concentrations in Figure 1 (3.9, 19.4, 38.8 mg/mL) was applied (3 µL) to the grids and the specimens were plunge frozen in liquid ethane just above its melting point temperature (91-93K) (3) using a Vitrobot Mark III (ThermoFisher) with settings 100% humidity, 4°C, blot time of 4 s, blot force 0, delay time 0).

Single-particle data collection and processing. The apoferritin dataset was collected over a one-day session using a 300 keV transmission electron microscope (TFS Titan Krios G4, LMB Krios 4) equipped with a direct electron detector (Falcon4i) and a cold FEG. All data processing was done using Relion 5.0 (6, 7). The movie stack and gain references were imported for motion correction using MotionCorr2 (8) and CTF estimation using CTFFind4.1 (9). Micrographs were then culled to remove over-focused/off-target and others of insufficient quality, reducing the number collected from 586 to 466 which were used for further processing. Particles were first picked with the Laplacian-of-Gaussian picker, extracted with downsampling twice and subjected to 2D classification. The best classes were used as an input for template-based picking. Picked particles were initially downsampled twice during extraction and subjected to several rounds of 2D classification. The initial map used for 3D refinement was an apoferritin structure determined earlier and low-pass filtered to 20 Å. Particles were subjected to multiple rounds of 3D classification and re-extracted without downsampling. The final particle set (114,010 particles) was refined in 3D several times, including rounds of CTF refinement to correct for the anisotropic magnification, per-micrograph and per-particle defocus, astigmatism, B-factor and phase shift, as well as higher-order aberrations, and Bayesian polishing.

Tomographic analysis of particle distributions. Tilt series were collected using a 300 keV transmission electron microscope (TFS Titan Krios G3, LMB Krios 2) equipped with a direct electron detector (Falcon4i). A dose-symmetric tilt scheme was used (10), starting from 0 tilt with a range (−45, 45°) or (−60, 60°C) and a tilt increment of 3°. Images were collected in the EER electron counting format with 4 $e^-/Å^2$ dose per tilt. The resulting tilt series were imported into Relion 5.0 TOMO software and motion-corrected using MotionCorr2 (8). CTF fitting estimation was performed using CTFFIND4.1 (9). The tilt series were then manually edited to exclude off-target shots. The tilt series were aligned using IMOD's patch-tracking alignment (patch size 100 nm, overlap 50%) and reconstructed in Relion TOMO. The resulting tomogram in .mrc format was imported into Fiji2 and the plugin CLIJ2 (11) was used to obtain maximum X/Y or Z projections presented in the figures. For the on-the-grid particle concentration calculation, particles were counted manually with a clicking tool using the maximum Z projection image. The 3D project function with an angle increment of 1° was used to measure the thickness in pixels of the vitrified specimen. As the reconstructed specimen plane might be slightly tilted, the tilt angle was also measured and the final result was corrected for this angle. Each thickness/angle measurement was taken at least three times to estimate the error. The tomographic concentration of the particles was calculated from the volume of the layer and the manually counted number of apoferritin particles with the known molecular weight of the complex.

Movie S1. High-speed camera recording of the full cycle of spraying and plunging for one clipped grid with a frame rate of 1.25 ms/frame

References

1. Schindelin J, et al. (2019) Fiji: an open-source platform for biological-image analysis. *Nat Methods* 9(7):676–682.
2. Wagner T, Hiner M, Xraynaud (2017) Ridge detection 1.4.0. *Zenodo*.
3. Russo CJ, Scotcher S, Kyte M (2016) A precision cryostat design for manual and semi-automated cryo-plunge instruments. *Review of Scientific Instruments* 87:114302.
4. Danev R, Yanagisawa H, Kikkawa M (2021) Cryo-EM performance testing of hardware and data acquisition strategies. *Microscopy* 70(6):487–497.
5. Schaffner W, Weissmann C (1973) A rapid, sensitive, and specific method for the determination of protein in dilute solution. *Analytical Biochemistry* 56:502–514.
6. Scheres SH (2012) RELION: Implementation of a Bayesian approach to cryo-EM structure determination. *Journal of Structural Biology* 180(3):519–530.
7. Burt A, et al. (2024) An image processing pipeline for electron cryo-tomography in RELION-5. *FEBS Open Bio* 14:1788–1804.
8. Zheng SQ, et al. (2017) MotionCor2: anisotropic correction of beam-induced motion for improved cryo-electron microscopy. *Nature Methods* 14:331–332.
9. Rohou A, Grigorieff N (2015) CTFFIND4: Fast and accurate defocus estimation from electron micrographs. *Journal of Structural Biology* 192:216–221.
10. Hagen WJ, Wan W, Briggs JA (2016) Implementation of a cryo-electron tomography tilt-scheme optimized for high resolution subtomogram averaging. *Journal of Structural Biology* 197:191–198.
11. Haase R, et al. (2020) CLIJ: GPU-accelerated image processing for everyone. *Nature Methods* 17(1):5–6.
